# Metabolic Reprogramming in Resting and Activated Immune Cells

**DOI:** 10.4172/2153-0769.1000188

**Published:** 2017-03-10

**Authors:** H Sun, X Li

**Affiliations:** Department of Pathology and Laboratory Medicine, Perelman School of Medicine, University of Pennsylvania, PA 19104, USA

**Keywords:** Glutaminolysis, Metabolic programming, Bio-precursors

## Abstract

Immune cell activation and proliferation are closely linked to their metabolic programming. These activated immune cells share many features with tumor cells and are capable to respond to stimulations quickly and reprogram their metabolism to fight with invading pathogens. The corresponding changes in metabolism provide immune cells with energy and bio-precursors to match with necessity of immune functions. The major metabolic pathways utilized by immune cells for the purpose of protecting body from invading pathogens are glycolysis, glutaminolysis, fatty acid synthesis and oxidation, and mitochondria oxidative phosphorylation. These pathways play crucial roles in immune cell activation and differentiation. In this review, we describe how immune cells engage in certain metabolic processes according to their functional needs with a focus on T cells and macrophages.

## Introduction

In steady stage, the cells in immune system are relatively quiescent with minimal activities and survival needs but they possess the ability to quickly respond to pathogens or environmental challenges and constantly orchestrate their effector functions [1]. Once pathogens are detected in the body, the cells will switch to an activated stage and become to fight with possible inflammation, infection, and/or any attacks from environment. The shaping from a quiescent stage to an activated stage requires energy and bio-precursor. Recent studies indicate that metabolism and immune cell functions are closely linked [2]. The change in metabolism not only passively supports the activation status, but also crucially influences the differentiation of immune cells. To understand how these fundamental processes influence each other may provide novel treatments for inflammatory and autoimmune diseases.

## Cells of the immune system

Defense against invading infectious pathogens and noninfectious foreign substances is mediated by innate immunity and adaptive immunity. Innate immune responses are early reactions of immune system and carried out by innate immune cells including macrophages, neutrophils, and natural killer cells. They provide the first defense barrier and response of protection while adaptive immune responses offer more specific actions to the pathogens. There are two types of adaptive immune responses: antibody-based humoral immunity and cell-mediated cellular immunity. Humoral immunity is carried out by B-lymphocytes, also called B cells and provides specific and specialized attack to extracellular microbes and their toxin, facilitates the elimination of these pathogens. Cellular immunity by T-lymphocytes (also called T cells) with the function functions to eliminate intracellular microbes, such as virus and some bacteria residing inside host cells. The principal cell types in immune system consist of innate immune cells, T and B-lymphocytes, antigen-presenting cells, and effector cells.

## Major Metabolic Pathways Exploited by Immune Cells

The major metabolic pathways that immune cells use to generate energy and biosynthesis materials are glycolysis, tricarboxylic acid cycle, oxidative phosphorylation, fatty acid metabolism, and glutaminolysis.

### Glycolysis

Glycolysis is a process that breaks down glucose and forms pyruvate with the production of two molecules of ATP. The pyruvate end product of glycolysis can be used in either anaerobic respiration if no oxygen is available or in aerobic respiration via the TCA cycle which yields much more usable energy for the cell.

### The TCA cycle

The generated pyruvate from glycolysis has two fates. One is to import into the mitochondria and to be converted to acetyl-CoA, entering the TCA cycle to produce NADPH and FADH_2,_ which can be used to generate ATP in mitochondria.

The alternative fate of pyruvate is to be converted to lactate in the cytosol that is an important strategy to regenerate NAD^+^ under hypoxic conditions. The generated NAD^+^ can be used in the process of generating pyruvate from glucose.

### Oxidative phosphorylation’s in mitochondria

This is a process occuring in mitochondria that NADH and FADH_2_ generated from glycolysis and TCA cycles are passed along electron transport chain to oxygen and generate usable energy ATP.

### Fatty acid metabolism

Fatty acid metabolism includes two parts: one is catabolic process by which fatty acid is broken down by in mitochondria to generate Acetyl-CoA, which enters TCA cycle and NADH/FADH_2,_ which are used in oxidative phosphorylation to produce ATP. The other process is anabolic which is opposite to fatty acid oxidation, which is to creat fatty acid using Acetyl-CoA through actions of fatty acid synthase. This process is carried out during cytoplasm.

## Metabolic Pathways T cell Activation and Proliferation

**Figure 1:**
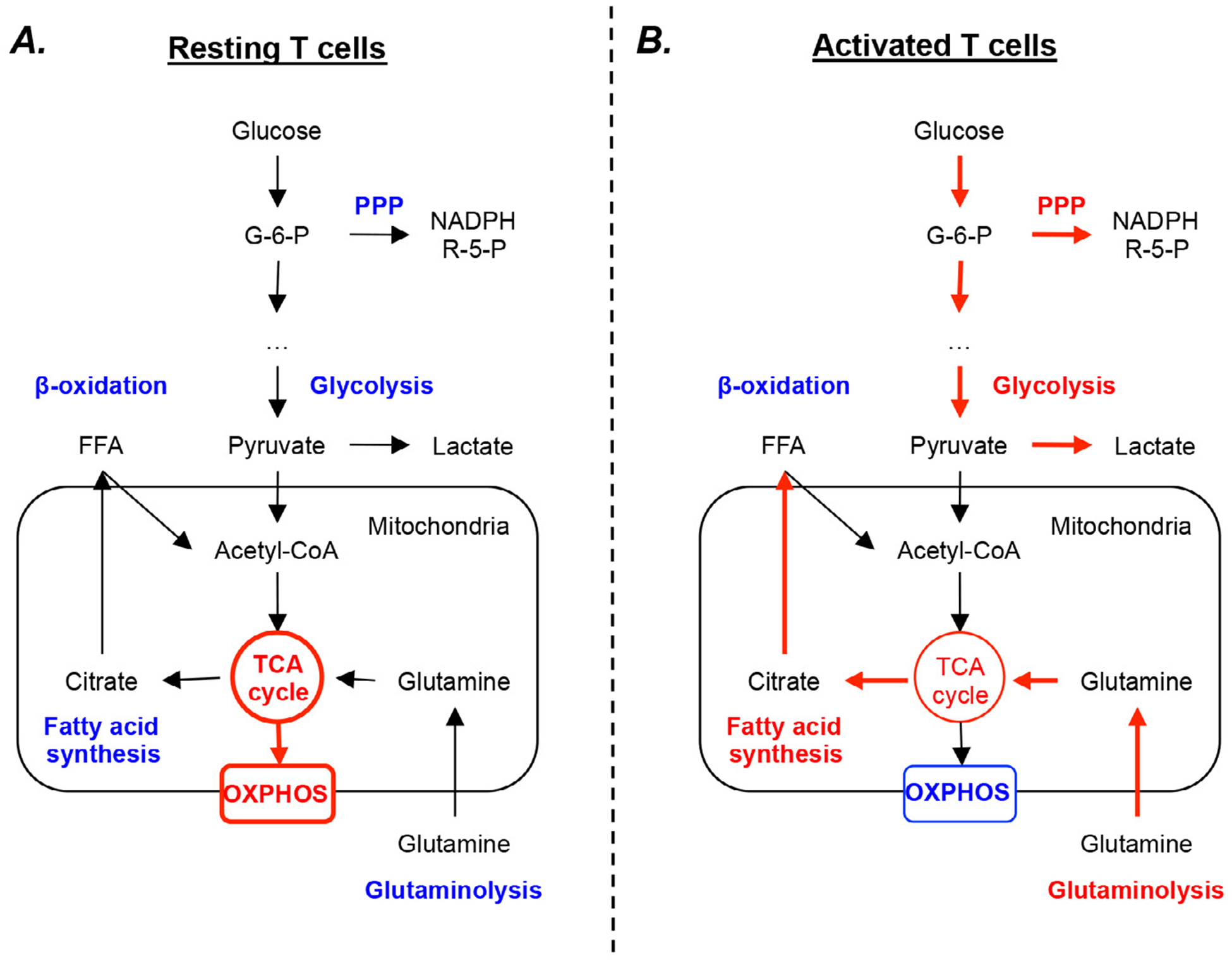
Metabolic features of resting and activated T cells. A) At steady state, T cells mainly rely on mitochondrial oxidative phosphorylation (OXPHOS) process using the reducing equivalents generated from tricarboxylic acid (TCA) cycle. B) When activated, T cells rapidly upregulate their glycolysis, glutaminolysis, pentose phosphate (PPP), and fatty acid synthesis pathways, and downregulate their TCA cycle rate. Blue letters and arrows indicate pathways with low activity, while red letters and arrows indicate dominant pathways. G-6-P, glucose-6-phosphate; NADPH, nicotinamide adenine dinucleotide phosphate; R-5-P, ribose-5-phosphate.

Before T cells encounter any antigens and/or danger signals presented to them by antigen presenting cells (APC), they remain quiesent metabolically. In this state, naïve cells use IL-7 to maintain its homeostatic proliferation and survival, with a metabolical level similar to memory T cells [3–5]. They don’t undergo massive clonal expansion and cytokine secretion; the requirement for energy and bio-precursors is minimal; therefore, they use available corbons mainly from glucose by glycolysis in mitochondria via oxidative phosphorylation and fatty acid by β-oxidation, to generate ATP to maintain homeostatic growth and survival (Figure 1a).

Upon antigen and other danger signal stimulation, naïve T cells become highly proliferative and differentiated. They undergo massive clonal expansions and secret a large amount of cytokines, and differentate into Th1, Th2, Th17, and Treg cells in case of CD4 cells and cytotoxic T lymphocytes (CTL) in case of CD8 cells. The demands for energy and building blocks are very different from that of naïve T cells and require metabolic reprogramming [6]. The proliferating T cells mostly employ aerobic glycolysis(also called Warburg effect) [7,8] while keeping TCA cycle low to support the robust growth and proliferation by providing bio-precursors needed for nucleotide, animo acid, and lipid synthesis [9,10]. Aerobic glycolysis is a process that glucose is metabolized to lactate instead of Acetyl-coA in the presence of oxygen. Althrough the process provides less ATP, it provides fast fuels and the carbon source for biosynthesis (Figure 1b).

Besides glycolysis, glutaminolysis is also indispensable for T cell activation and proliferation for the purpose of supplying a fue source and biosynthetic precursors for protein and nucleic acid biosynthesis. When activated, T cells consume glutamine at a comparable rate to that of glucose [9]. During glutaminolysis, the amino acid is converted to pyruvate, glutamate, and other metabolic intermediates serving as building blocks for protein synthesis, fatty acid sythesis, and nucleotide synthesis and redox control [11,12].

Although less is known about lipid metabolism in T cell prolifeation and function, more and more evidence showed the importance and correlation between fatty acid metabolism and T cell proliferation. Firstly, it was reported that T cells switch to fatty acid synthesis from fatty acid oxidation upon activation and proliferation [13]. Secondly, Miguel and colleagues also found that T cell proliferation ability is closely correlated with the membrane lipid composition [14]. However, the mechanism of the connection remains largely unknown.

## Metabolic Pathways in Innate Cell Responses

Macrophages undergo profound metabolic reprogramming in response to danger signal or cytokine stimulation. These metabolic changes not only provide energy and biosynthesis power, but also directly regulate macrophage effector functions. In response to proinflammatory stimulation such as Lipopolysacharide (LPS), macrophages shift from oxidative phosphorlation and fatty acid oxidation toward glycolysis and fatty acid synthesis [15]. The significance of this phenomenon, termed the Warburg effect, which usually occurs in cancerous cells or highly proliferative cells (such as activated T cells), is poorly defined in macrophages since they usually do not proliferate after inflammatory stimulation. Recent studies, however, shed light into the importance of the Warburg effect in activated macrophages.

Firstly, it was found that macrophages stimulated with LPS utilized the Warburg effect to accumulate citrate, an intermediate in the cytric acid cycle. The accumulated citrate then contributes to the production of Nitric Oxide (NO), Reactive Oxygen Species (ROS), and prostaglandins, all of which play significant proinflammatory roles in LPS-activated macrophages [16]. Secondly, in LPS-stimulated macrophages, the accumulation of succinate, which is another cytric acid cycle intermediate, mediates the production of proinflammatory cytokine Interleukin 1β (IL-1β) [17]. This is due to the fact that the accumulated succinate could activate hypoxia inducible factor 1 alpha subunit (HIF1α), which then transcriptionally induces IL-1β gene expression. In addition, the increased mitochondrial oxidation of succinate drives mitochondrial ROS production. As a result, LPS-stimulated macrophages shift their mitochondrial metabolism from ATP production towards ROS production that promotes a proinflammatory state [18].

## Future Perspectives

In this review, we dicussussed the metabolic pathways immune cells adopt corresponding to their status and function a particular focus on T cells and macrophages. Although an increased and still increasing literature has enriched our understanding of the connections and switches between these two equally complicated processes, still a lot remains unknown. The biggest question is whether the metabolism change itself can serve as a trigger or signal to initiate downstream signalling pathways. The second question is how metabolism affects gene regulation in immune cells and from what levels. It is evident now that glycose and lipid metabolism can profoundly shape immune cell functions. Therefore, understanding the molecular pathways of these processes may offer a novel therapeutical treatment for inflammatory diseases.
